# Assessment of Atmospheric Pressure Plasma Treatment for Implant Osseointegration

**DOI:** 10.1155/2015/761718

**Published:** 2015-05-19

**Authors:** Natalie R. Danna, Bryan G. Beutel, Nick Tovar, Lukasz Witek, Charles Marin, Estevam A. Bonfante, Rodrigo Granato, Marcelo Suzuki, Paulo G. Coelho

**Affiliations:** ^1^Department of Biomaterials and Biomimetics, New York University, 345 East 24th Street, Room 804 S, New York, NY 10010, USA; ^2^School of Chemical Engineering, Oklahoma State University, 423 Engineering North, Stillwater, OK 74078, USA; ^3^Department of Prosthodontics, University of São Paulo Bauru College of Dentistry, Al Otávio Pinheiro Brisola 9-75, 17012-901 Bauru, SP, Brazil; ^4^Department of Prosthodontics, Tufts University School of Dental Medicine, 1 Kneeland Street, Boston, MA 02111, USA; ^5^Department of Periodontology and Implant Dentistry, New York University College of Dentistry, New York, USA; ^6^Division of Engineering, New York University Abu Dhabi, Abu Dhabi, UAE

## Abstract

This study assessed the osseointegrative effects of atmospheric pressure plasma (APP) surface treatment for implants in a canine model. Control surfaces were untreated textured titanium (Ti) and calcium phosphate (CaP). Experimental surfaces were their 80-second air-based APP-treated counterparts. Physicochemical characterization was performed to assess topography, surface energy, and chemical composition. One implant from each control and experimental group (four in total) was placed in one radius of each of the seven male beagles for three weeks, and one implant from each group was placed in the contralateral radius for six weeks. After sacrifice, bone-to-implant contact (BIC) and bone area fraction occupancy (BAFO) were assessed. X-ray photoelectron spectroscopy showed decreased surface levels of carbon and increased Ti and oxygen, and calcium and oxygen, posttreatment for Ti and CaP surfaces, respectively. There was a significant (*P* < 0.001) increase in BIC for APP-treated textured Ti surfaces at six weeks but not at three weeks or for CaP surfaces. There were no significant (*P* = 0.57) differences for BAFO between treated and untreated surfaces for either material at either time point. This suggests that air-based APP surface treatment may improve osseointegration of textured Ti surfaces but not CaP surfaces. Studies optimizing APP parameters and applications are warranted.

## 1. Introduction

Osseointegration, the direct structural assimilation of bone to an implant, is a topic of particular importance to orthopaedic surgeons. In arthroplasty (e.g., around the prosthesis) and in trauma (e.g., around screws), bony anchorage onto the implant surface can make the difference between success and failure of reconstructive surgery. Surface modifications may increase the osseointegrative properties of implants [1–3], and optimizing bony ingrowth has been the subject of extensive investigation literature for years [4–8].

Osseointegration is postulated to proceed by adsorption of proteins, which then recruit osteoprogenitor cells onto the implant surface [6, 9, 10]. Cellular adhesion may be enhanced by manipulation of the implant's surface properties (e.g., charge, texture, and polarity) to yield a hospitable microenvironment on the implant surface [11–13]. Chemical (e.g., oxidation [14] and plasma treatment [4, 15]) and topographical (e.g., particulate coating, pressure blasting, and chemical abrasives) modifications have all been investigated as potential modifications [16–18]. Additionally, surface energy, a measure of unbonded surface atoms, is often examined to ascertain a material's ability to facilitate osseointegration [9, 19]. High surface energy states foster cellular adhesion [20]. Surface energy may be divided into nonpolar (disperse) components and polar components, which accounts for polar groups, electric charges, and free radicals, as well as the roughness of the surface [21].

The focus of this study is the utilization of plasma treatment on the implant's surface to increase osseointegration. Plasma treatment may be utilized as an agent to alter a surface's properties in a variety of ways. Plasma may be generated with heat, termed thermal plasma treatment, often at low pressures [4]. Understandably, this presents environmental challenges such as achieving appropriate temperature and pressure for processing, limiting the utility of the procedure to industrial settings. Another technique for plasma generation occurs at ambient temperatures and atmospheric pressures, termed atmospheric pressure plasma (APP) treatment. In this process, argon gas has been described as an energy carrier, promoting the formation of reactive compounds on the implant surface [4]. Prior studies have demonstrated enhanced osseointegration through argon-based APP treatment [4, 19, 22, 23]. However, investigations concerning the effect of other gases, especially compressed air that is readily available in operatories, are warranted if the ultimate goal is large-scale utilization of APP for increasing the osseointegration of implantable devices.

In the present study, we investigated the utilization of compressed air as an alternative to argon. As a safe, portable, and cost-effective technology, air-based APP treatment is thought to improve surface energy of implant surfaces by the removal of debris or molecules that may have become adsorbed during processing. Specifically, we evaluated air-based APP treatment on two widely used surfaces, namely, textured titanium (Ti, obtained by grit-blasting/acid-etching procedures) and calcium phosphate- (CaP-) coated implants, comparing treated to untreated surfaces in a canine radius model.

## 2. Materials and Methods

### 2.1. Physicochemical Evaluation

The textured Ti surfaces were obtained through grit-blasting/acid-etching (Integra-Ti, Bicon LLC, Boston, MA) of plateau root form endosseous Ti-6Al-4V bulk alloy implants of 3.5 mm in diameter by 8 mm in length. CaP-coated (Integra-CP, Bicon LLC, Boston, MA) bulk alloy implants were also used in this study. The experimental set of implants was treated immediately prior to implantation with an APP application of compressed air for a total of 80 seconds (20 seconds per implant quadrant). The control groups were left untreated. Previous detailed physical/chemical characterization [24, 25] of these surfaces has shown that the CaP coatings were ~20–30 *μ*m in thickness and presented ~40% crystalline HA content. Surface roughness assessment has also shown that the plasma-sprayed hydroxyapatite surface roughness was higher (1.8 ± 0.25 *μ*m) than the grit-blasted/acid-etched surfaces (0.66 ± 0.10 *μ*m) [24, 25].

The plasma was applied with a KinPen (INP, Greifswald, Germany) device (length = 190 mm, diameter = 20 mm, and weight = 170 g). The KinPen was used for the generation of a plasma jet at atmospheric pressure connected to a high-frequency power supply (1.5 MHz, 2–6 kV peak-to-peak, system power 230 V, 65 W). The air supply was connected to a gas controller (Multi Gas Controller 647C, MKS Instruments, Andover, MA), which was set to flow at 5 standard liters per minute (l pm) [19]. The compressed air composition was the same as regular atmospheric composition at 16% oxygen, 1% hydrogen, and 78% nitrogen.

Physicochemical characterization was performed on nine implants from each group. The surface morphology was observed by scanning electron microscopy (SEM, Philips XL 30, Eindhoven, Netherlands) at 5000x magnification and an acceleration voltage of 15–20 kV (*n* = 3 per group).

The surface energy was assessed using the Owens-Wendt-Rabel-Kaelble method [26]. A micropipette (OCA 30, Data Physics Instruments GmbH, Filderstadt, Germany) was used to deposit 0.5 mL droplets of distilled water (DI-H_2_O), ethylene glycol (C_2_H_6_O_2_), and diiodomethane (CH_2_I_2_) onto the surface of a member of each implant group. Software SCA30 (version 3.4.6, build 79) captured and analyzed each image. Each type droplet was applied on the apical flat surface of the plateau root form implants (*n* = 3).

Surface chemical characterization was performed by X-ray photoelectron spectroscopy (XPS) at three different surface spots along the implant length. Three implants from each group were degassed to 10^−7 ^torr and transferred under vacuum to a Kratos Axis 165 multitechnique XPS spectrometer (Kratos Analytical, Chestnut Ridge, NY). Survey and high-resolution spectra were obtained using a 165 mm mean radius concentric hemispherical analyzer operated at a constant pass energy of 160 eV for survey and 80 eV for high-resolution scans (take-off angle of 90°, spot size of 150 *μ*m × 150 *μ*m).

### 2.2. *In Vivo* Model

For the* in vivo *study, seven adult male beagle dogs (number determined based on previous studies [4, 19, 22, 23]), approximately 1.5 years of age, were used. The experimental protocol received the approval of the École Nationale Vétérinaire d'Alfort (Maisons-Alfort, Val-de-Marne, France). NIH guidelines for the care and use of laboratory animals and policies of the Animal Welfare Act were observed. The beagles remained in the facility for an approximate two-week acclimation period prior to any surgical intervention.

All surgical procedures were performed under general anesthesia. Intramuscular (IM) atropine sulfate (0.044 mg/kg) and xylazine chlorate (8 mg/kg) were administered for preanesthesia. General anesthesia was then obtained following an IM injection of ketamine chlorate (15 mg/kg). Following skin preparation, a 5 cm incision was made and the deeper tissues were dissected to expose the diaphysis of the radius.

Four implants (untreated Ti, APP-treated Ti, untreated CaP, and APP-treated CaP) were placed along each radius. The implants were press fit into 3.5 mm drill holes made with a power drill (1200 rpm for the pilot hole, 800 rpm for sequentially larger bits) under continuous saline irrigation. They were placed from proximal to distal directions, approximately 1 cm apart, in various positions in the sequence in order to eliminate any confounding of the results based on implant location* in vivo*. At the initial surgery, implants were placed only in the left limb to be analyzed six weeks postoperatively. Three weeks later, implants for a three-week postoperative assessment were placed in the right limb in a second procedure.

Closure was achieved with standard layered suturing techniques with VICRYL 4-0 (Ethicon Johnson, Miami, FL) for deep tissues and nylon 4-0 (Ethicon Johnson, Miami, FL) for skin. The dogs remained in the animal care facility and received antibiotics (benzyl penicillin benzathine 20.00 IU/kg) and anti-inflammatory medication (ketoprofen 1%, 1 mL/5 kg) for pain control. Euthanasia was carried out by anesthesia overdose six weeks after the first surgical procedure.

### 2.3. Histomorphologic Evaluation

At necropsy, the radii with implants were retrieved by sharp dissection. The bone blocks were kept in 10% buffered formalin solution for 24 hours, washed in running water for 24 hours, and gradually dehydrated in a series of alcohol solutions ranging from 70 to 100% ethanol. Following dehydration, the samples were embedded in a methacrylate-based resin (Technovit 9100, Heraeus Kulzer GmbH, Wehrheim, Germany) according to the manufacturer's instructions. The blocks were then cut into slices (~300 *μ*m thick) centering the implant along its long axis with a precision diamond saw (Isomet 2000, Buehler Ltd., Lake Bluff, IL) and glued to acrylic plates with an acrylate-based cement (Technovit 7210 VLC, Heraeus Kulzer GmbH, Wehrheim, Germany), and a 24-hour setting time was allowed prior to grinding and polishing. The sections were then reduced to a final thickness of ~30 *μ*m by means of a series of silicon carbide (SiC) abrasive papers (280, 400, 800, 1200, and 2500; Buehler Ltd., Lake Bluff, IL) in a grinding/polishing machine (Metaserv 3000, Buehler Ltd., Lake Bluff, USA) under water irrigation. The sections were then stained with toluidine blue and subjected to optical microscopy for histomorphologic evaluation.

The histologic features were evaluated at 50x–200x magnification (Leica DM2500M, Leica Microsystems GmbH, Wetzlar, Germany). The bone-to-implant contact (BIC) was determined by computer software (Leica Application Suite, Leica Microsystems GmbH, Wetzlar, Germany). The length of bone along the implant perimeter was measured and divided by e total implant perimeter to calculate the BIC percentage. The bone area fraction occupancy (BAFO) between plateaus was determined at 100x magnification with the same microscope and software by subtracting the percentage area occupied by bone from the total available area within the healing chambers.

### 2.4. Statistics

Statistical analysis was performed with SPSS (version 19, New York, NY). XPS data were evaluated by one-way ANOVA at 95% level of significance. For all outcomes concerning the animal study, statistical significance was set to a 95% level of confidence and the number of dogs was considered the statistical unit for all comparisons. For the histomorphometric-dependent variables BIC and BAFO, a GLM ANOVA (general linear model) was employed including surface group and time* in vivo* as independent variables (surgical site position was preliminarily evaluated and, due to a lack of effect on BIC and BAFO, was ultimately excluded from further analysis).

## 3. Results

### 3.1. Surface Characterization

**Figure 1:**
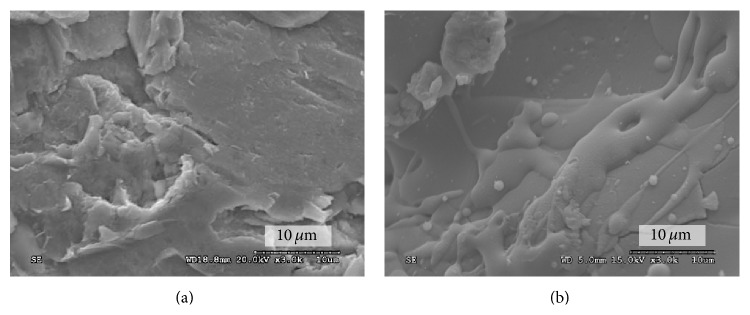
Scanning electron micrographs of (a) titanium (Ti) and (b) calcium phosphate (CaP) implant surfaces before atmospheric pressure plasma (APP) treatment.

**Figure 2:**
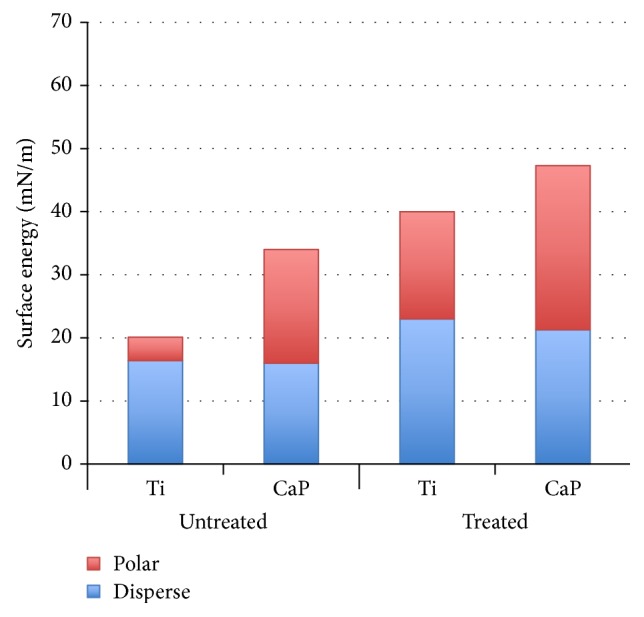
Surface energy bar graph for polar and disperse components of the titanium (Ti) and calcium phosphate (CaP) groups before (untreated) and after (treated) atmospheric pressure plasma treatment.

The SEM micrographs of the implant surfaces revealed a grit-blasted textured surface (Figure 1(a)) on the Ti implant and a textured microstructure surface on the CaP implant (Figure 1(b)). The surface energy assessment showed a substantial increase of approximately 15 mN/m in the polar component and a small increase (5 mN/m) of the disperse component of the Ti implant group immediately after plasma treatment (Figure 2). Both the polar and disperse components of the CaP implant group showed increases (9 mN/m and 5 mN/m, resp.) following the plasma application.

The XPS survey analysis of the control and air APP-treated implants showed peaks of Ti and O for both treated and untreated Ti surfaces. High-resolution spectrum evaluation demonstrated that for both surfaces carbon (C) was observed primarily as hydrocarbon (C–C, C–H) with lower levels of oxidized C forms. For the untreated Ti surfaces, XPS detected the atomic percent values (mean ± SD) of 45.0 ± 5.1 for C, 1.5 ± 0.5 for aluminum (Al), 0.1 ± 0.2 for nitrogen (N), 14 ± 4.7 for Ti, and 37.0 ± 3.6 for O (Table 1). Vanadium (V) was detected at trace levels. Compared to the control group, the air APP-treated Ti surfaces showed decreased levels of C (38.0 ± 4.7) and increased levels of Ti (19.0 ± 3.9) and O (43.0 ± 4.3). Similar values were observed for Al, N, and V in the control and experimental groups.

For the CaP surfaces, the XPS survey analysis of the implant surface showed peaks of calcium (Ca), C, O, and phosphate (P) for both the control and air APP-treated samples. High-resolution spectrum evaluation revealed that for both surfaces C was observed primarily as hydrocarbon with lower levels of oxidized C forms. For both the treated and untreated groups, Ca and P were detected in varied atomic concentrations. For the CaP group, the atomic percent values (mean ± SD) were 38.0 ± 4.2 for C, 42.0 ± 5.2 for O, 11.0 ± 2.5 for Ca, and 7.0 ± 1.3 for P (Table 1). When compared to the control CaP group, the APP-treated CaP implants presented increases in O (48.0 ± 3.1), Ca (12.0 ± 2.3), and P (10.0 ± 2.4) atomic percent levels. A decrease in C content was observed at 22.0 ± 4.5 atomic percent.

### 3.2. *In Vivo* Model

The animal surgical procedures and follow-up demonstrated no complications or other clinical concerns. Therefore, no implant was excluded due to clinical instability, which was clinically determined after euthanization.

**Figure 3:**
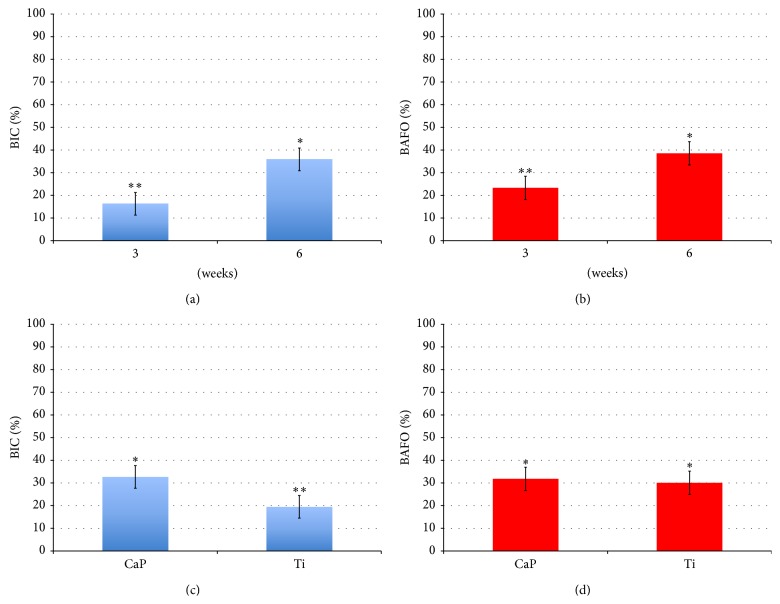
*In vivo* bone and implant characteristics as a function of ((a) and (b)) time* in vivo* collapsed over surface type and ((c) and (d)) implant surface type collapsed over time* in vivo*. BIC: bone-to-implant contact; BAFO: bone area fraction occupancy; Ti: titanium; and CaP: calcium phosphate. Asterisks indicate statistically homogeneous groups (different numbers of asterisks depict that groups were statistically different at *P* < 0.05) assessed for *n* = 7 animals.

**Figure 4:**
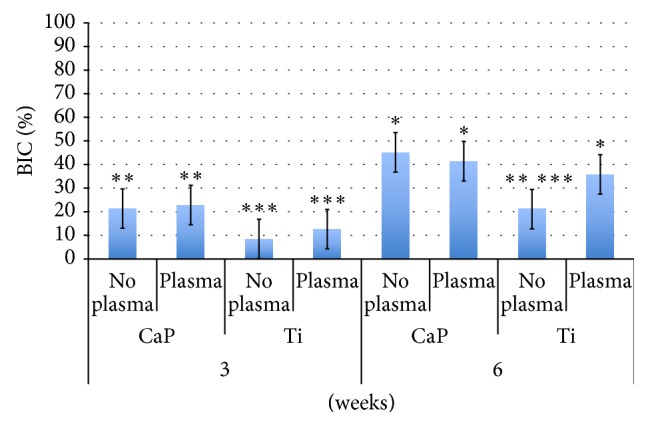
Osseointegration of implants by bone-to-implant contact (BIC). Mean values with standard deviation bars are provided for the untreated (no plasma) and atmospheric pressure plasma-treated (plasma) titanium (Ti) and calcium phosphate (CaP) groups at respective experimental time periods. Asterisks indicate statistically homogeneous groups (different numbers of asterisks depict that groups were statistically different at *P* < 0.05) assessed for *n* = 7 animals.

**Figure 5:**
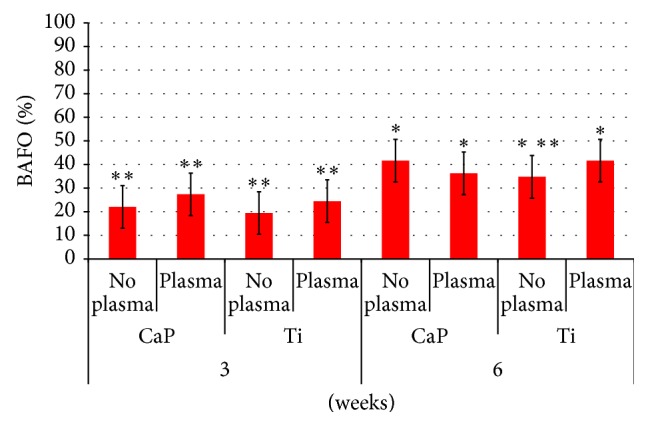
Osseointegration of implants by bone area fraction occupancy (BAFO). Mean values with standard deviation bars are provided for the untreated (no plasma) and atmospheric pressure plasma-treated (plasma) titanium (Ti) and calcium phosphate (CaP) groups at respective experimental time periods. Asterisks indicate statistically homogeneous groups (different numbers of asterisks depict that groups were statistically different at *P* < 0.05) assessed for *n* = 7 animals.

The BIC results as a function of time* in vivo* and implant surface presented significant differences (both *P* < 0.001) (Figure 3). No difference between the treated and untreated groups of either the CaP or Ti surface was observed at three weeks (Figure 4). A significant (*P* < 0.001) difference was observed in BIC for the Ti implant but not for the CaP one at six weeks. BAFO measurements did not show a significant (*P* = 0.57) difference between the treated and untreated groups (Figure 3). However, there was a significant difference in BAFO between 3 and 6 weeks* in vivo* (*P* < 0.001) (Figure 3). No differences in BAFO were noted between any groups at either three or six weeks* in vivo* (Figure 5).

**Figure 6:**
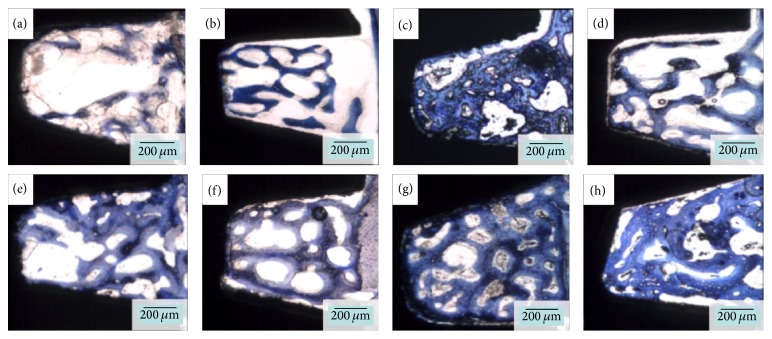
Representative overview of the histological micrographs. Untreated calcium phosphate (CaP) implants at three (a) and six (e) weeks, respectively; untreated titanium (Ti) implant at three (b) and six (f) weeks, respectively; atmospheric pressure plasma- (APP-) treated CaP implants at three (c) and six (g) weeks; and APP-treated Ti group at three (d) and six (h) weeks.

Qualitative evaluation of the stained histological sections of the untreated groups after three weeks* in vivo* demonstrated minimal woven bone in proximity to the implant surface (Figures 6(a) and 6(b)). Their APP-treated counterparts, however, presented a more well-distributed bone formation within the plateau and a higher degree of bone formation in proximity to the implant surface (Figures 6(c) and 6(d)). At six weeks, the untreated surfaces presented lower degrees of bone formation in proximity to the implant surface (Figures 6(e) and 6(F)) relative to their APP-treated counterparts (Figures 6(g) and 6(h)). Such differences were more pronounced between the untreated and the APP-treated Ti surfaces. An overall large increase in bone formation occurred for all groups from 3 to 6 weeks.

## 4. Discussion

Endosseous implant surfaces have evolved from presenting the as-machined turned surface towards textured Ti surfaces (obtained by additive or subtractive engineering methods) under the unequivocal support that osseoconduction of textured surfaces is substantially improved relative to smoother surfaces [5, 11]. Recent research has also convincingly demonstrated that calcium- and phosphate-based coatings on Ti surfaces further increase the osseoconductivity obtained through texturing Ti surfaces alone [5, 11]. A plethora of* in vivo* laboratory models, including both surfaces evaluated in the present study, have shown that, regardless of the animal model, the plasma-sprayed CaP surface presents higher degrees of measurable osseointegration parameters and biomechanical fixation at early implantation times* in vivo* [27–29].

While the plasma spraying of CaP coatings is performed under controlled atmosphere and high temperatures in order to coat Ti implants with a 20–50 *μ*m thickness and is thus an industrial process that requires safety measures [5, 11], the APP technology evaluated in the present study has been previously utilized to surface treat implants of varied compositions and textures immediately prior to their implantation [4, 19, 22, 23]. APP usually operates at room temperature, substantially lower temperatures compared to the coating processing of hot plasma (at several thousand degrees). Thus, APP's ability to deliver surface modification [30] at room temperature through portable equipment is a promising technology for facilitating early osseointegration of any biocompatible implant surface regardless of its chemistry and topography. The characteristics of the technology make it more affordable and conducive to use in the operating room immediately prior to implant placement [4, 19, 22, 23]. It must be noted that other methods have been attempted to decrease surface contamination while increasing its energy. For instance, Buser et al. [31] have shown that increased levels of osseointegration can be obtained through an industrial surface cleaning and packaging method in a liquid phase relative to controls. Different from the proprietary process described by Buser et al. [31], a photofunctionalization method that can be utilized in the operating room immediately prior to implantation has been extensively described by Ogawa et al. [32–34] at both* in vitro* and* in vivo* settings. Both methods have demonstrated efficiency in increasing osseointegration levels.

Previous studies have used the KinPen device supplied with argon gas and indicated that the treatment enhanced osseointegration at early time points* in vivo* [4, 19, 22, 23]. Of these, some have assessed the same Ti and CaP surfaces utilized in the present study, and while over a 300% increase in osseointegration was obtained for argon-based APP-treated Ti surfaces relative to controls at three weeks* in vivo* [4], a much smaller size effect of approximately 80% was observed for the argon-based APP-treated CaP surfaces relative to their untreated counterparts at the same implantation time [22]. From both studies regarding physicochemical characterization, the increase in surface energy was more remarkable for the Ti surfaces compared to the CaP surfaces, and such relative differences between argon APP-treated and untreated surfaces are thought to be the reason for the relative differences in osseointegration.

The present study investigated whether exchanging argon for compressed air in the APP application furnished the same osseointegrative benefits. Relative to previous studies, both physicochemical and* in vivo* results depicted that compressed air APP treatment did increase surface energy and facilitated earlier osseointegration relative to controls, but such increases were not as remarkable as the results obtained with argon gas [4, 22]. The surface energy and XPS results showed that surface elemental chemistry was modified by the air-based APP treatment and that this change resulted in a higher degree of exposure of the surface chemical elements, mainly at the expense of the removal of adsorbed C species immediately after plasma treatment [35]. For both Ti and CaP surfaces, increases were noted but were not nearly as high as previously reported for argon gas [4, 22]. A limitation of the current investigation along with previous work [4, 22] is that no assessment of surface texture modification at the micrometer and nanometer scale was made after the different APP regimens were performed, thereby warranting further assessment of this important surface parameter.

The histological evaluation revealed that the interaction between tissue and implant surface occurred at three weeks* in vivo* for both experimental groups (APP-treated CaP and APP-treated Ti), while the untreated CaP and Ti groups displayed lower degrees of bone formation in proximity to the implant surface interaction until six weeks* in vivo*. Overall, the CaP implant surface did not benefit from the APP treatment, while increased bone-to-implant contact at 6 weeks was observed for the APP-treated Ti surface. Comparing the current study to previous studies [4, 22] of argon-based NTP treatment, the air-based plasma did not increase the surface energy to the same degree as argon. Additionally, the carbon present on the implant surface did not decrease to the same extent as it had with the argon APP treatment. Thus, given the previous and current results, it can be concluded that the air-based APP treatment is not as effective as its argon-based counterpart.

## 5. Conclusions

While air-based APP treatment did not surpass argon-based treatment, the results of this study do not invalidate its utility either. It would be of interest to investigate the technology's impact on bone formation when longer treatment times are employed. Additionally, assessment of the biomechanical integrity of the bone-implant construct would provide information about the quality of formed bone, which could be considered in addition to this study's quantitative assessment.

## Figures and Tables

**Table 1:** X-ray photoelectron spectroscopy spectra for titanium (Ti) and calcium phosphate (CaP), both untreated and atmospheric pressure plasma- (APP-) treated. Mean (SD) values are provided.

Chemical element (%)	Ti surface		CaP surface	
	Untreated	APP-treated	Untreated	APP-treated
Al2p	1.5 (0.5)	∗∗	∗∗	∗∗
C1s	45.0 (5.1)	38.0 (4.7)	38.0 (4.2)	22.0 (4.5)
N1s	0.1 (0.2)	—	—	—
Ca2p	—	—	11.0 (2.5)	12.0 (2.3)
O1s	37.6 (3.6)	43.0 (4.3)	42.0 (5.2)	48.0 (3.1)
P2p	—	—	7.0 (1.3)	10.0 (2.4)
Ti2p	14.0 (4.7)	19.0 (3.9)	∗∗	∗∗
V2p3	∗∗	∗∗	∗∗	∗∗

Al: aluminum; C: carbon; N: nitrogen; Ca: calcium; O: oxygen; P: phosphate; V: vanadium.

^**^Only trace amounts were present.
